# Noncommunicable diseases and injuries and health surveys

**DOI:** 10.1590/1980-549720230001.supl.1

**Published:** 2023-04-21

**Affiliations:** I Universidade Federal de Minas Gerais, School of Nursing, Department of Maternal and Child Nursing and Public Health – Belo Horizonte (MG), Brazil.; II Instituto Brasileiro de Geografia e Estatística – Rio de Janeiro (RJ), Brazil.

Noncommunicable diseases and injuries represent the greatest cause of morbidity and mortality in Brazil and worldwide, besides resulting in premature deaths, disabilities, loss of quality of life, and important economic impacts^1,2^. Noncommunicable diseases and injuries comprise two major groups of events: chronic noncommunicable diseases (NCDs), mainly characterized by cardiovascular diseases, chronic respiratory diseases, neoplasms, and diabetes mellitus; and external causes such as accidents and violence^1,2^.

NCDs are estimated to cause 41 million deaths worldwide per year, which is equivalent to more than 70% of deaths, of which 15 million are premature (<70 years)^2^. In Brazil, in 2019, the overall mortality due to NCDs corresponded to about 76% and premature deaths totaled 66.1%^3^. NCDs result in devastating consequences for individuals, families, and communities in addition to overloading healthcare systems^4^. Violence and accidents are responsible for over 4.8 million deaths worldwide; in Brazil, there are about 150 thousand deaths each year, including lives lost prematurely in addition to disabilities, loss of quality of life, and important impacts on the Brazilian Unified Health System (SUS) and the economy^5^.

This supplement from the Brazilian Journal of Epidemiology (*Revista Brasileira de Epidemiologia* – RBE) addresses the challenge of health surveys and information systems in the monitoring of noncommunicable diseases and injuries. National surveys are components of extreme relevance in a national health system and essential to know the health profile, the distribution of risk factors, their trends, health inequalities, and social determinants. The information periodically collected enables the monitoring of health actions and programs in different population strata in addition to contributing to subsidize public policies^6^.

The supplement highlights the importance of the National Survey of Health (*Pesquisa Nacional de Saúde* – PNS), generating key information to monitor the health of Brazilians. One of the articles analyzes data from the PNS laboratory, collected in the years 2014/2015 and the reference intervals referring to the blood cell count of Brazilian adults, with and without sickle cell trait^7,8^. The database of the PNS becomes innovative by incorporating the collection of biological material (blood and urine) in a subsample, making it fundamental to evaluate NCDs, the occurrence of diseases, and the distribution of biological markers in the population. The inclusion of the “PNS laboratory” component in the next edition of the research is of great relevance^6^.

In another article concerning PNS, work-related and commuting accidents among employed Brazilians in 2013 and 2019 were analyzed^9^. The PNS enables to measure these occurrences, which are underreported, due to a large part of the working population being inserted in informal work, without basic rights. PNS shows important inequalities in the occurrence of work-related accidents, which predominate among men, young people, Black individuals, those with lower levels of education, and rural workers^9^.

For the first time, in the PNS 2019, population data on violence against gay, lesbian, and bisexual (LGB+) people were analyzed in Brazil. The study points out that the reported prevalence values are about two to three times higher among LGB+, showing the vulnerability of the LGB+ population in the country and the need for social protection policies^10^.

Articles analyze the database of the National Survey of School Health (*Pesquisa Nacional de Saúde do Escolar* – PeNSE), conducted by the Brazilian Institute of Geography and Statistics (IBGE), in partnership with the Brazilian Ministry of Health, with the aim of estimating the prevalence of risk factors and health protection of schoolchildren aged 13 and 17 years throughout the country as well as tracking trends over time. Reis et al.^11^ highlight the importance of parental supervision regarding risky sexual behavior among Brazilian adolescents, pointing out that young people with higher number of parental supervision indicators simultaneously had higher prevalence of condom use^11^. Another study analyzes child labor, which is part of contexts of vulnerability and socioeconomic inequalities in which children and adolescents are inserted, perpetuating cycles of vulnerabilities. The authors highlight that child labor is associated with higher prevalence of risk factors, which may result in NCDs throughout life^12^. Another article analyzes the demand, as well as the use, by Brazilian adolescents for health services, according to PeNSE, showing the importance of Primary Health Care in meeting this demand^13^.

Two articles analyze data from the Surveillance System of Risk and Protective Factors for Chronic Diseases by Telephone Survey (Vigitel), which annually monitors, since 2006, via telephone interviews, the risk and protection factors of NCDs in adults (age ≥18 years) living in the capitals of the 26 Brazilian states and the Federal District. One of the articles analyzes the trend of behaviors among older adults^14^; the other analyzes the trend of indicators of physical activity in adults, highlighting the worsening of indicators in the COVID-19 pandemic years, with reduced physical activity in spare time and increased sedentary behavior^15^.

Behaviors also changed among adolescents, according to the *ConVid Adolescentes – Pesquisa de Comportamentos* (Research on Adolescents’ Behavior), which analyzes the reduction of alcohol consumption among adolescents during the COVID-19 pandemic. This consumption is associated with the presence of friends, “their peers,” which was greatly reduced during social distancing in the period^16^.

In this supplement of the RBE, one of the articles discusses the mortality trends of NCDs, showing the decline in rates over the last decade and the importance of correcting mortality data from the Brazilian Mortality Information System (*Sistema de Informação sobre Mortalidade* – SIM), especially in the redistribution of garbage codes (GC), aiming at the adequacy of estimates^17^. Another article also uses corrections by GC and analyzes mortality patterns due to external causes in municipalities in the mining region of Vale do Paraopeba, before the rupture of the Brumadinho dam, in the state of Minas Gerais, being a baseline in the region, prior to the environmental disaster^18^.

The information produced in this supplement demonstrates the health conditions of Brazilians and promotes scientific communication, by the publication of articles that present original results of analyses related to the Noncommunicable Diseases and Injuries and Health Surveys topic. The information periodically collected by these studies allows monitoring health actions and programs in different population strata and contributes to subsidize public policies. We wish you all a good reading of these articles.
