# Supplementary material for: Noncommunicable diseases and injuries and health surveys
Source: Rev Bras Epidemiol. 2023 Apr 21;26(Suppl 1):e230001. doi: 10.1590/1980-549720230001.supl.1 (PMC10176727; doi:10.1590/1980-549720230001.supl.1)
Supplement: Translation

# Translation: Doenças e agravos não transmissíveis e inquéritos em saúde

**DOI:** 10.1590/1980-549720230001.supl.1.1

**Affiliations:** I Universidade Federal de Minas Gerais, Escola de Enfermagem, Departamento de Enfermagem Materno Infantil e Saúde Pública – Belo Horizonte (MG), Brasil.; II Instituto Brasileiro de Geografia e Estatística – Rio de Janeiro (RJ), Brasil.

As doenças e agravos não transmissíveis (DANT) representam a maior causa de morbimortalidade no Brasil e no mundo, além de resultarem em mortes prematuras, incapacidades, perda da qualidade de vida e importantes impactos econômicos^1,2^. As DANT compreendem dois grandes grupos de eventos: as doenças crônicas não transmissíveis (DCNT), caracterizadas, principalmente, por doenças cardiovasculares, pelas doenças respiratórias crônicas, neoplasias e *diabetes mellitus*; e as causas externas, tais como os acidentes e as violências^1,2^.

Estima-se que as DCNT sejam responsáveis por 41 milhões de mortes no mundo ao ano, o que equivale a mais de 70% dos óbitos, dos quais 15 milhões são prematuros (<70 anos)^2^. No Brasil, em 2019, a mortalidade geral por DCNT correspondeu a cerca de 76% e os óbitos prematuros somaram 66,1%^3^. As DCNT resultam em consequências devastadoras para indivíduos, famílias e comunidades, além de sobrecarregarem os sistemas de saúde^4^. As violências e os acidentes são responsáveis por mais de 4,8 milhões de mortes no mundo; no Brasil, são cerca de 150 mil mortes a cada ano, incluídas as vidas perdidas prematuramente, além de incapacidades, perda da qualidade de vida e importantes impactos no Sistema Único de Saúde (SUS) e na economia^5^.

Este suplemento da *Revista Brasileira de Epidemiologia* aborda o desafio dos inquéritos e sistemas de informações em saúde no monitoramento das DANT. Os inquéritos de abrangência nacional são componentes de extrema relevância em um sistema nacional de saúde e essenciais para conhecer o perfil de saúde, a distribuição dos fatores de risco, suas tendências, as desigualdades em saúde e os determinantes sociais. As informações coletadas periodicamente permitem o monitoramento de ações e programas de saúde em distintos estratos populacionais, bem como contribuem para subsidiar as políticas públicas^6^.

O suplemento destaca a importância da Pesquisa Nacional de Saúde (PNS), gerando informações fundamentais para monitorar a saúde do brasileiro. Um dos artigos analisa dados do laboratório da PNS, coletados nos anos de 2014/2015 e os intervalos de referência referentes ao hemograma de adultos brasileiros, com e sem traço falciforme^7,8^. A base da PNS torna-se inovadora pela incorporação da coleta de material biológico (sangue e urina) em uma subamostra, tornando-se fundamental para avaliar as DCNT, a ocorrência de doenças e a distribuição dos marcadores biológicos na população. Apresenta-se como de grande relevância a inclusão do componente “laboratório da PNS” na próxima edição da pesquisa^6^.

Em outro artigo da PNS foram analisados os acidentes no deslocamento e no trabalho entre brasileiros ocupados em 2013 e 2019^9^. A PNS possibilita mensurar essas ocorrências, que são subnotificadas, em função de grande parte da população trabalhadora se inserir em trabalhos informais, sem direitos básicos. A PNS mostra importantes desigualdades na ocorrência dos acidentes de trabalho, que predominam entre homens, jovens, pretos, com menor escolaridade, trabalhadores da zona rural^9^.

Pela primeira vez, na PNS 2019, foram analisados dados populacionais sobre a violência contra pessoas gays, lésbicas e bissexuais (LGB+) no Brasil. O estudo aponta que as prevalências referidas são cerca de duas a três vezes mais elevadas entre LGB+, mostrando a vulnerabilidade da população LGB+ no país e a necessidade de políticas de proteção social^10^.

Artigos analisam a base de dados da Pesquisa Nacional de Saúde do Escolar (PeNSE), realizada pelo Instituto Brasileiro de Geografia e Estatística (IBGE), em parceria com o Ministério da Saúde, com o objetivo de estimar as prevalências dos fatores de risco e proteção à saúde de escolares entre 13 e 17 anos em todo o país, bem como acompanhar as tendências ao longo do tempo. Reis et al.^11^ destacam a importância da supervisão dos pais quanto ao comportamento sexual de risco entre adolescentes brasileiros, apontando que jovens com maior número de indicadores de supervisão parental simultaneamente apresentaram maiores prevalências do uso de preservativos^11^. Outro estudo analisa o trabalho infantil, que se insere em contextos de vulnerabilidade e desigualdades socioeconômicas nos quais crianças e adolescentes estão inseridos, perpetuando ciclos de vulnerabilidades. Os autores destacam que o trabalho infantil se associa com prevalências mais elevadas de fatores de risco, podendo resultar em DCNT ao longo da vida^12^. Outro artigo analisa a procura, bem como a utilização, por serviços de saúde por parte de adolescentes brasileiros, segundo a PeNSE, mostrando a importância da Atenção Primaria no atendimento dessa demanda^13^.

Dois artigos analisam dados da Vigilância de Fatores de Risco de Doenças Crônicas Não Transmissíveis (Vigitel), que monitora, anualmente, desde 2006, por meio de entrevista telefônica, os fatores de risco e proteção de DCNT em adultos (idade ≥18 anos) residentes nas capitais dos 26 estados brasileiros e do Distrito Federal. Um dos artigos analisa a tendência de comportamentos entre idosos^14^, o outro analisa a tendência de indicadores de atividade física em adultos, destacando a piora dos indicadores nos anos da pandemia, com redução da atividade física em tempo livre e aumento do comportamento sedentário^15^.

Os comportamentos também foram alterados entre adolescentes, é o que mostra a pesquisa ConVid Adolescentes — Pesquisa de Comportamentos, que analisa a redução do consumo de bebidas alcoólicas entre adolescentes durante a pandemia de COVID-19. Esse consumo associa-se à presença de amigos, “os pares”, o que foi bastante reduzido durante o distanciamento social no período^16^.

Neste suplemento da RBE, um dos artigos aborda as tendências de mortalidade das DCNT, mostrando o declínio das taxas ao longo da última década e a importância da correção dos dados de mortalidade do Sistema de Informação sobre Mortalidade (SIM), em especial na redistribuição dos códigos *garbage* (CG), visando a adequação das estimativas^17^. Outro artigo também utiliza as correções por CG e analisa os padrões de mortalidade por causas externas em municípios da região mineradora do Vale do Paraopeba, antes do rompimento da barragem de Brumadinho, Minas Gerais, sendo uma linha de base na região, previamente ao desastre ambiental^18^.

As informações produzidas neste suplemento revelam as condições de saúde dos brasileiros e promovem a comunicação científica, por meio da publicação de artigos que apresentam resultados originais de análises referentes ao tema Doenças e Agravos Não Transmissíveis e Inquéritos em Saúde. As informações coletadas periodicamente nestas pesquisas permitem o monitoramento de ações e programas de saúde em distintos estratos populacionais, bem como contribuem para subsidiar as políticas públicas. Desejamos a todos boa leitura destes artigos.
